# Barriers and facilitators in diagnosing axial spondyloarthritis: a qualitative study

**DOI:** 10.1007/s00296-024-05554-z

**Published:** 2024-03-12

**Authors:** Charles A. Hay, Jon Packham, James A. Prior, Christian D. Mallen, Sarah Ryan

**Affiliations:** 1 School of Medicine, Keele University, Keele, UK; 2 Academic Unit of Population and Lifespan Sciences, University of Nottingham, Nottingham, UK; 3 Midlands Partnership University NHS Foundation Trust, Stafford, UK; 4 School of Nursing and Midwifery, Keele University, Keele, UK

**Keywords:** Axial spondyloarthritis, Ankylosing spondylitis, Diagnosis, Qualitative

## Abstract

**Introduction:**

Diagnosis of axial spondyloarthritis (axSpA) is frequently delayed for years after symptom onset. However, little is known about patient and healthcare professional (HCP) perspectives on barriers and facilitators in axSpA diagnosis. This study explored the experiences and perceptions of both groups regarding the factors affecting the timely diagnosis of axSpA.

**Method:**

Semi-structured interviews with patients with axSpA and axSpA-interested HCPs from the United Kingdom (UK) were performed by telephone or Microsoft Teams and focussed on the individuals’ perspective of the diagnostic journey for axSpA. Interview transcripts were thematically analysed.

**Results:**

Fourteen patients with axSpA (10 female, 4 male) and 14 UK based HCPs were recruited, the latter comprising of 5 physiotherapists, 4 General Practitioners, 3 rheumatologists, a nurse, and an occupational therapist. Barriers to diagnosis identified by patients and HCPs were: difficult to diagnose, a lack of awareness, unclear referral pathways, patient behaviour and patient/HCP communication. Patient-identified facilitators of diagnosis were patient advocacy, clear referral processes and pathways, increased awareness, and serendipity. HCPs identified promoting awareness as a facilitator of diagnosis, along with symptom recognition, improvements to healthcare practice and patient/HCP communications.

**Conclusion:**

Poor communication and a lack of understanding of axSpA in the professional and public spheres undermine progress towards timely diagnosis of axSpA. Improving communication and awareness for patients and HCPs, along with systemic changes in healthcare (such as improved referral pathways) could reduce diagnostic delay.

**Supplementary Information:**

The online version contains supplementary material available at 10.1007/s00296-024-05554-z.

## Introduction

Axial spondyloarthritis (axSpA) is an autoimmune inflammatory arthritis predominantly affecting the sacroiliac joints and spine, sometimes with additional articular and extra-articular involvement [1]. AxSpA is commonly characterised by chronic low back pain, fatigue, and reduction in mobility/physical function [1], all of which can impact on physical, psychological, and social wellbeing, including work roles and personal relationships [2].

Delay in the diagnosis of axSpA is common, typically between 2 and 5 years globally [3]. Such delay can result in more progressive disease, reduced response to treatment and poorer patient outcomes [2, 4]. Although many population-level [3] and electronic health record [5–8] studies have tried to identify factors associated with delayed axSpA diagnosis, there is still limited understanding of the factors that contribute to this delay [9].

Several studies have investigated aspects of the axSpA diagnostic journey and their effect on and interplay with diagnostic delay from the perspectives of the patient or healthcare professionals’ (HCPs) [10–14]. Results concurred that levels of awareness and understanding of axSpA in the public and healthcare spheres were lacking, and systemic healthcare problems and difficulties diagnosing the disease existed. However, only one study was based in the United Kingdom (UK), with its distinct healthcare structure and did not collect information from HCPs [10], a key partner in supporting the patient to achieve a final diagnosis. Furthermore, Martindale et al. did not primarily examine the effect on delay of aspects of the diagnostic process but explored delay as a modifier and intensifier of issues encountered along the diagnostic journey.

To consider how delay can be reduced, it is necessary to identify and fully understand the challenges involved in the diagnostic process. This study explored the experiences and perceptions of patients with axSpA and HCPs regarding the barriers and facilitators affecting the timely diagnosis of axSpA.

## Methods

The theoretical framework underpinning this work is phenomenology [15]. Phenomenological research is focussed on understanding the meaning of an experience from an individual’s perspective [16]. The use of semi-structured interviews aided the exploration of diagnostic delay by enabling the participants to share their experiences. The reporting of this study is based on the Consolidated Criteria for Reporting Qualitative Health Research (COREQ) [17] (Supplementary Table 1). Ethical approval was obtained from the NHS Health Research Authority on 11/08/2020 (IRAS project ID 262371).

Participants were identified based on the following criteria: ≥ 18 years of age from across the UK, with a diagnosis of axSpA after 2009, the year that the Assessment of Spondyloarthritis International Society (ASAS) axSpA classification criteria were introduced [18, 19]. ASAS is a widely accepted classification criteria for axSpA and underpins the UK National Institute for Health and Care Excellence (NICE) guidelines for diagnosis [20]. Participants were recruited through multiple routes, including a local rheumatology centre, social media (Twitter, now X) [21] and a national patient charity (National Axial Spondyloarthritis Society (NASS)) [22]. Diagnosis was self-reported. Possible participants were excluded if they possessed cognitive impairments which would preclude the level of conversational involvement required by this study. In Addition, potential participants were excluded if they did not possess a level of comprehension and use of English. HCPs working in the UK who had experience in the management of patients with axSpA were recruited opportunistically through a snowball sampling technique beginning with known contacts within local organisations. To be included in the study, HCPs had to have previously been involved in the diagnosis, management, or treatment of axSpA in the NHS. In addition to a lack of exposure to axSpA, the only other exclusion criterion was if they were unavailable to take part in the study during the study timeframe.

All participants took part in one semi-structured interview with the same interviewer (CH) between August 2020 and March 2021. The interviewer undertook the interviews during his PhD studies and had received qualitative research training from Keele University and Oxford University; he had no prior relationship with the patient interviewees, but a proportion of the HCP participants were previously known. Patients with axSpA were interviewed via telephone and HCPs were interviewed via telephone/Microsoft Teams. The interviews were digitally recorded, transcribed verbatim and pseudo-anonymised. Separate topic guides were produced for the patient and HCP interviews (Supplementary file 2). Patients with a diagnosis of axSpA from the local NASS group (*n* = 6) formed our Patient and Public Involvement and Engagement (PPIE) group. The PPIE members assisted in the development of the topic guides, along with other patient facing documentation, such as information sheets and consent forms. Both topic guides focussed on participants’ experience, knowledge, and perceptions of the journey to axSpA diagnosis and went through an iterative process of drafting, acting on suggested edits and comments from our PPIE members.

### Analysis

Thematic analysis [23], undertaken using NVivo12 (QSR International) [24], was used as an exploratory method by which patient and HCP experiences and perceptions of the diagnostic journey could be understood. Within-participant analysis was undertaken by two members of the research team (CH & SR) before looking for patterns across participants. Each transcript was read repeatedly to ensure familiarisation with the data and to identify codes, themes, and categories from the interviews. The method of analysis was based on that described by Braun and Clarke [25], iterated upon in 2019, and described in full in Supplementary File 3.

Information power [26] (a means of attempting to judge the likelihood that sample size and data-collection quality are sufficient to deliver results which substantially answer a research question) was judged based on the characteristics of the present study (aim of the study, sample specificity, use of theory, quality of dialogue and analysis strategy). Comparing with recent studies which interviewed patients [10] and GPs [12], the target sample sizes of this current study had credible information power.

## Results

Fourteen patients with axSpA from the UK were recruited (ten female, four male). Median age was 43 years (range 24–59), median age of first symptom onset was 20 years (range 11–30), median age of diagnosis was 39.5 years (range 20–53) and the median delay to diagnosis was 15.5 years (range 1–20 years) (Supplemental 4). Patient interviews ranged between 50 min and 1 h 12 min. Fourteen UK-based HCP were recruited, comprising of 5 physiotherapists, 4 GPs, 3 rheumatologists, a nurse, and an occupational therapist. The frequency with which HCPs interacted with patients with known/suspected axSpA, ranged widely from 3 to 280 per year (Supplemental 5). HCP interview length ranged from 39 min to 1 h 35 min.

### The patient perspective of barriers to diagnosis

Five themes were identified from patient interviews regarding barriers of diagnosis: patient/HCP interactions, the difficulties in diagnosing axSpA, patient behaviour, lack of public and healthcare awareness of axSpA and sub-optimal practice in healthcare. Each theme and its related data are described in Table 1.

**Table 1:** The patient perspective of barriers to diagnosis

Theme	Brief description	Sub-themes	Example quotes
Patient/HCP interaction	Barriers caused by issues with the interaction between patients and the HCPs with whom they consult	Patient communication	“I’m angry at myself for not having made more of a fuss…the AS has got worse over the last couple of years and I'm thinking, 'Why didn't I say something six months ago or a year ago?'” (P010) “… so I’ve got this pain that I’ve had for a few years in the lower back and now I’ve hurt my upper back as well… so for three years before then I already knew I’d had it… I’ve never complained profusely enough to the GP to get it looked at if you know what I mean.” (P033)
		HCP communication	“… I was being completely dismissed and made to feel like I was overreacting… I just felt disbelief… like you’re banging your head against a brick wall.” (P021) “It was very much a, ‘go away. What more do you want us to do?…’” (P004)
AxSpA is difficult to diagnose	Barriers caused by the complexity of axSpA diagnosis	Not presenting in the classical way/unclear and inconsistent symptoms	“I suppose that it didn’t help that I didn’t probably present in the classic way with I’ve gone in with a bad back that’s lasted three months… it’s with it starting higher up… because my joints are kind of more affected in the beginning. With my knee first up… I think that was probably all against me when it came to diagnosis.” (P002) “It would come, and it would go. You know pain would return and I would think, ‘oh that’s weird, I don’t think I did anything to make that pain come back’, and then it would disappear again.” (P032)
		Alternative explanations for symptoms	“I was very, very sporty and so I did have various aches and pains, but I always put it down to playing rugby or whatever… I was a bit obsessive, so I figured I’d damaged myself.” (P010) “I was screaming in pain with pains in my legs – and I was told I had growing pains…” (P030) “… when I was about 25, it was the first bout of really bad back pain I had when I was pregnant and… of course it was blamed on the pregnancy… basically that was it. ‘You’re pregnant, it’s sciatica, baby’s laying…’”
		HCPs missed symptoms suggestive of axSpA	“… my MRIs lit up like a Christmas tree with inflammation but nothing on the x-rays. No, you know, evidence of bone-fusing, so that was what was delaying everything…” (P035) “… manifested into different things, which again are pointers to AS and again nobody picked it up… nothing was flagged with it or anything…” (P025)
		Missed opportunities for diagnosis	“… if they’d just scanned that little bit lower, they’d have seen I had scoliosis further down and sclerosis… which is one of the first major obvious things in AS I believe.” (P002) “… [my rheumatologist] looked through the notes and said, ‘to be honest, I don’t think there’s much point me doing anything else… I can see your entire history and I can’t believe you’ve not made it here before.’” (P004)
Patient behaviour	Patient behaviour and personality traits slowing down journey to axSpA diagnosis	Patients’ acceptance of their symptoms	“I mean at the time I just put it down to it was just one of those things that happens to people but yeah, you’ve just got to get on with life, I suppose.” (P004) “I’ve always been in pain for years and years and you just get on with it, don’t you? You have to. You’ve got a family. You’ve got things you need to try and do” (P018)
		Low confidence in healthcare	“I just gave up on it… I’d go to the GP, my back was still hurting, so they’d send me for more steroids, they never looked at why my back was hurting… I just thought there’s no point in complaining any more…” (P033) “I'm not going to bother with the GP because all they do is give me painkillers. I could have gone back earlier. In my mindset, I think I closed off the GP route.” (P024)
Lack of awareness of axSpA	Limited awareness of axSpA in healthcare and general public spheres reduces likelihood of suspicion of inflammatory arthritis being raised by symptoms	Patient and public lack of awareness	“[By the time I was diagnosed] all my joints had swollen up, my hands, my feet… I couldn’t believe all my joint pains were related to this bad back I’d had for years…” (P002) “When I got diagnosed, I had no idea what it was. My family hadn’t heard about it. My friends had no idea what it was.” (P025)
		Lack of awareness in healthcare system	“I mean I’m sure my first doctor had probably never even heard of it…” (P015) “I don’t think it ever occurred to any GP even when I hit 50 and all me joints had swollen up I don’t think it ever occurred to them.” (P002)
Sub-optimal practice in healthcare	Configuration of, and practice within, healthcare services slowing down journey to diagnosis	Lack of defined referral pathway	“I think there needs to be more access to.. the referral to a rheumatologist… it can take somebody years just to get that referral because there are a lot of GP’s who are reluctant to do so…” (P033) “…20 years’ worth of back and forwarding…” (P015)
		Lack of communication/co-ordination between different healthcare services	“There doesn’t seem to be an umbrella department that says the eye people should talk to the rheumatology should talk to the GP… There’s nothing, no branch connecting all these things and yet all of the symptoms are connected as far as I can see.” (P003) “I think it would have been easier for them if it was the same doctor, they would be picking it up ‘cause I don’t think doctors have got time to sit and read through everybody’s history before that patient walks through the door.” (P025)
		Insufficient consultation time	“… I don’t think your quick five minutes or ten minutes they allocated are enough to go over everything you need… Because we’re so used to hiding our pain, getting to talk about it and to say exactly what’s going on takes time sometimes.” (P002) “… a 10-min visit if you’re lucky and it’s a mad rush in and back out again. You trying to remember everything you say and you come back and you forget half of what you wanted to say…” (P018)

#### Patient/HCP interaction

The majority of patients described communication with HCPs as a barrier to diagnosis. Patients felt they had been overly deferential in consultation and had not sought recognition that they may have something more serious than was being implied. They found HCPs could be dismissive of the symptoms they were experiencing.

#### AxSpA is difficult to diagnose

For many patients the difficulty in diagnosing their disease led to delayed diagnosis. Many attributed the presentation of their symptoms as a reason for their axSpA being difficult to diagnose, suspecting that if early symptoms were not predominantly around the lower back or pelvis, then any suspicion for axSpA was not raised until later in the disease development. Symptoms were often also labelled as being caused by strain and sports injury, which led to a lack of suspicion of the disease. In Addition, even when patients were investigated using imaging, their results were sometimes not sufficiently conclusive to lead naturally towards a diagnosis of axSpA.

#### Patient behaviour

Two aspects of patient behaviour which influenced the delay in diagnosis were stoicism regarding symptoms and low confidence in healthcare services to manage their condition. Patients’ tendency to ignore symptoms was partially influenced by previous unsatisfactory consultations with HCPs.

#### Lack of awareness of axSpA

Delay was perceived to be related to the lack of general awareness of axSpA both by the public and healthcare professionals. This encompassed an incomplete understanding of the disease, its characteristics, and its impact. Some female patients, for example, were told their symptoms were not axSpA because their GP was under the incorrect apprehension that axSpA is a predominantly male disease.

#### Sub-optimal practice in healthcare

The configuration of healthcare services and the duration of consultations was seen as sub-optimal, with the process of referral to a rheumatologist often perceived to be a cause of delay. Referral was a slow and complex process with ill-defined steps and outcomes, which often resulted in the patient being reviewed by different specialities with little rationalisation. Many patients found consultations were too short to explain their symptoms and this reduced the likelihood of symptom recognition occurring and the possible diagnosis of axSpA being made.

### The patient perspective of facilitators of diagnosis

Five themes related to facilitators of diagnosis were identified from patient interviews, these were: patient behaviour and advocacy, patient characteristics, good practice in healthcare, education and awareness, and serendipity (Table 2).

**Table 2:** The patient perspective of facilitators of diagnosis

Theme	Brief description	Sub-themes	Example quotes
Patient behaviour and advocacy	Aspects of patient behaviour and advocacy of patients that speed up diagnosis	N/A	“… I thought, ‘Do you know what? I’m not going to home’… eventually I get admitted into the surgical ward. Got a CT scan the next day… and this young consultant, ‘I know what’s wrong with you’. I went, ‘Oh, don’t tell me it’s a kidney stone’, and he went, ‘No’, and he called it, he went, ‘It’s called sacroiliitis’” (P018) “… my mother and father were fighting to get people to just find an answer and they contacted my GP about it and that’s how I first got involved with [HCP] at the [Hospital] and that’s how I got my diagnosis.” (P035)
Patient characteristics	Patient behaviour and personality traits speeding up journey to axSpA diagnosis	Triggers to consultation	“I was in agony, absolute agony. So that was probably the main turning point for me… obviously went to the doctors about that…” (P002) “… I was constantly waking up at 2am and watching TV because I couldn’t sleep because my back was hurting… I was thinking this is crazy because it shouldn’t be impeding your life that much. So I went to the GP and I guess that’s the point at which you would say the journey to diagnosis had started…” (P003)
		Point at which patients realised their symptoms were something severe	“I’d been complaining to my partner that my vision was a bit blurry in one of my eyes and when we were out at the weekend for a walk and thick fog and mist rolled in… I said to him, ‘that’s what my eye looks like.’ He looked at me and said, ‘that’s not right.’” (P010)
Good practice in healthcare	Instances where patients’ healthcare experiences accelerated their journey to diagnosis/aspects of healthcare that can be improved		“…in the hospital my experience was great, when my history was being taken it felt like the first time I’d properly been listened to… when she diagnosed and said I’m going to hand you to my colleague who specialises in this… I was part of this well-oiled machine…” (P030) “[My GP] referred me to rheumatology. Whether she thought it was AS, I don’t know at that stage. But she knew that it was a problem that could be identified at rheumatology or investigated.” (P003)
Education and awareness	Means of educating and raising awareness of axSpA	HCP awareness and education	“I’d want to make sure medical professionals were educated in all the symptoms and how it can present itself, so arthritis is at least put on the table sooner as a possibility of someone’s condition.” (P035) “I think [what is desperately needed is] undergraduate training. There needs to be some way of auditing the training that’s going on to make sure it’s up to date for GPs, rheumatologists and AHPs as well.” (P021)
		Patient and public awareness	“… I think just raising awareness of it in terms of the public as well, so the public are going in and saying, ‘could I have [axSpA]?’” (P021)
Serendipity	Several patients attributed their diagnosis to a single, seemingly arbitrary, circumstance or coincidence		“… my aunty… was sat at the dinner table with Christmas… There’s me moving my entire body to speak to somebody and she was like, ‘What on earth have you done?… Right, I want you to come into my clinic’… checking my neck and the movement (she) said after seeing me twice, ‘It could be [axSpA].’” (P004) “The only reason that she got the ball rolling is because her dad has got ankylosing spondylitis… she just went, ‘I think you’ve got a thing called AS. ….. Let me send you off for an MRI’, and she was the first person to know that there was a link to all this stuff and actually to check it out.” (P025)
Theme	Brief description	Sub-themes	Example quotes
Patient/HCP interaction	Barriers caused by issues with the interaction between patients and the HCPs with whom they consult	Patient communication	“I’m angry at myself for not having made more of a fuss…the AS has got worse over the last couple of years and I'm thinking, 'Why didn't I say something six months ago or a year ago?'” (P010) “… so I’ve got this pain that I’ve had for a few years in the lower back and now I’ve hurt my upper back as well… so for three years before then I already knew I’d had it… I’ve never complained profusely enough to the GP to get it looked at if you know what I mean.” (P033)
		HCP communication	“… I was being completely dismissed and made to feel like I was overreacting… I just felt disbelief… like you’re banging your head against a brick wall.” (P021) “It was very much a, ‘go away. What more do you want us to do?…’” (P004)
AxSpA is difficult to diagnose	Barriers caused by the complexity of axSpA diagnosis	Not presenting in the classical way/unclear and inconsistent symptoms	“I suppose that it didn’t help that I didn’t probably present in the classic way with I’ve gone in with a bad back that’s lasted three months… it’s with it starting higher up… because my joints are kind of more affected in the beginning. With my knee first up… I think that was probably all against me when it came to diagnosis.” (P002) “It would come, and it would go. You know pain would return and I would think, ‘oh that’s weird, I don’t think I did anything to make that pain come back’, and then it would disappear again.” (P032)
		Alternative explanations for symptoms	“I was very, very sporty and so I did have various aches and pains, but I always put it down to playing rugby or whatever… I was a bit obsessive, so I figured I’d damaged myself.” (P010) “I was screaming in pain with pains in my legs – and I was told I had growing pains…” (P030) “… when I was about 25, it was the first bout of really bad back pain I had when I was pregnant and… of course it was blamed on the pregnancy… basically that was it. ‘You’re pregnant, it’s sciatica, baby’s laying…’”
		HCPs missed symptoms suggestive of axSpA	“… my MRIs lit up like a Christmas tree with inflammation but nothing on the x-rays. No, you know, evidence of bone-fusing, so that was what was delaying everything…” (P035) “… manifested into different things, which again are pointers to AS and again nobody picked it up… nothing was flagged with it or anything…” (P025)
		Missed opportunities for diagnosis	“… if they’d just scanned that little bit lower, they’d have seen I had scoliosis further down and sclerosis… which is one of the first major obvious things in AS I believe.” (P002) “… [my rheumatologist] looked through the notes and said, ‘to be honest, I don’t think there’s much point me doing anything else… I can see your entire history and I can’t believe you’ve not made it here before.’” (P004)
Patient behaviour	Patient behaviour and personality traits slowing down journey to axSpA diagnosis	Patients’ acceptance of their symptoms	“I mean at the time I just put it down to it was just one of those things that happens to people but yeah, you’ve just got to get on with life, I suppose.” (P004) “I’ve always been in pain for years and years and you just get on with it, don’t you? You have to. You’ve got a family. You’ve got things you need to try and do” (P018)
		Low confidence in healthcare	“I just gave up on it… I’d go to the GP, my back was still hurting, so they’d send me for more steroids, they never looked at why my back was hurting… I just thought there’s no point in complaining any more…” (P033) “I'm not going to bother with the GP because all they do is give me painkillers. I could have gone back earlier. In my mindset, I think I closed off the GP route.” (P024)
Lack of awareness of axSpA	Limited awareness of axSpA in healthcare and general public spheres reduces likelihood of suspicion of inflammatory arthritis being raised by symptoms	Patient and public lack of awareness	“[By the time I was diagnosed] all my joints had swollen up, my hands, my feet… I couldn’t believe all my joint pains were related to this bad back I’d had for years…” (P002) “When I got diagnosed, I had no idea what it was. My family hadn’t heard about it. My friends had no idea what it was.” (P025)
		Lack of awareness in healthcare system	“I mean I’m sure my first doctor had probably never even heard of it…” (P015) “I don’t think it ever occurred to any GP even when I hit 50 and all me joints had swollen up I don’t think it ever occurred to them.” (P002)
Sub-optimal practice in healthcare	Configuration of, and practice within, healthcare services slowing down journey to diagnosis	Lack of defined referral pathway	“I think there needs to be more access to.. the referral to a rheumatologist… it can take somebody years just to get that referral because there are a lot of GP’s who are reluctant to do so…” (P033) “…20 years’ worth of back and forwarding…” (P015)
		Lack of communication/co-ordination between different healthcare services	“There doesn’t seem to be an umbrella department that says the eye people should talk to the rheumatology should talk to the GP… There’s nothing, no branch connecting all these things and yet all of the symptoms are connected as far as I can see.” (P003) “I think it would have been easier for them if it was the same doctor, they would be picking it up ‘cause I don’t think doctors have got time to sit and read through everybody’s history before that patient walks through the door.” (P025)
		Insufficient consultation time	“… I don’t think your quick five minutes or ten minutes they allocated are enough to go over everything you need… Because we’re so used to hiding our pain, getting to talk about it and to say exactly what’s going on takes time sometimes.” (P002) “… a 10-min visit if you’re lucky and it’s a mad rush in and back out again. You trying to remember everything you say and you come back and you forget half of what you wanted to say…” (P018)

#### Patient advocacy

Two forms of patient advocacy were proposed as a means towards facilitating a diagnosis of axSpA:

1. Self-advocacy, i.e. patients pushing for further investigation into their symptoms and not accepting what they felt to be unhelpful outcomes of consultation.
2. Advocacy by another, such as support in the consultation of a friend or family member, or by a charity or affiliated organisation.

#### Patients’ characteristics

Patients described the symptoms and circumstances which initiated a consultation including unbearable pain and the increasing impact of their symptoms on normal life, while others were encouraged to see a doctor by people around them who had become increasingly concerned. These events, described as triggers, started the patients’ process towards diagnosis.

#### Good practice in healthcare

Good practice in healthcare which assisted the process towards diagnosis included symptom awareness in HCPs and well executed history taking, leading to appropriate referral (as opposed to continuing unhelpful symptomatic treatment without referral).

#### Education and awareness

Patients perceived that improving the public visibility of axSpA would increase the likelihood of quicker diagnosis, as would education programs in healthcare. The resultant lower threshold for suspicion of pain being caused by inflammation, along with reduced ambiguity when discussing the disease could reduce a delay.

#### Serendipity

Several patients attributed their diagnosis to a single, seemingly arbitrary occurrence or coincidence, as opposed to a systematic journey through healthcare.

### The healthcare professional perspective of barriers to diagnosis

Five themes relating to barriers to diagnosis were identified in interviews with HCPs: axSpA being difficult to diagnose, the lack of public and healthcare awareness of axSpA, sub-optimal practice in healthcare, patient behaviour and characteristics, and patient/HCP interaction (Table 3).

**Table 3:** The healthcare professional perspective of barriers to diagnosis

Theme	Brief description	Sub-themes	Example quotes
axSpA is difficult to diagnose	Barriers caused by the complexity of axSpA diagnosis	Difficult to define and differentiate	“… lot of patients are picked up a bit late so it’s not uncommon to see people in their forties presenting with it. And I find that’s a more challenging group to tease out symptomology and I think the reasons for that might be because they have a mixed mechanical and inflammatory problem usually by that time” (H075) “I think it is just a tricky condition because there isn’t one single diagnostic test, it’s more forming the picture through a collection of signs, symptoms and your investigation results so it isn’t just any one thing.” (H080) “I know most women with AS tend to be misdiagnosed with fibromyalgia…” (H091)
		Investigations with uncertain outcomes	“… when you see a patient, you can’t just clinically say, this is axial spondyloarthritis’, you have to do imaging to confirm. That imaging can take time and sometimes you get equivocal imaging and then you have to repeat the imaging after a period of time and see how things evolve” (H072) “Especially the ones on the borderline of being diagnosed, but there’s not enough sort of from an investigation point of view to formally, formally diagnose them but you’ve got every clinical suspicion” (H071)
Lack of awareness of axSpA	Poor awareness of axSpA in healthcare and general public spheres reduces likelihood of suspicion of inflammatory arthritis being raised by symptoms	Patient and public lack of awareness	“… patients themselves lack awareness of inflammatory causes of back pain… They don’t know something like this exists, they just think it’s back pain, muscle strain, mechanical back pain, that sort of thing so that will often have delayed them seeking help” (H075) “… it’s not a common presentation, unless you’ve got someone in the family… I don’t think even Google brings up inflammatory back pain… they don’t usually come up with, ‘I think I’ve got axSpA.’” (H083)
		Lack of awareness in healthcare	“I think physiotherapists may not understand the relationship between back pain and the other associated sort of things that come along with spondyloarthropathy, so things like psoriasis, enthesitis, uveitis, inflammatory bowel disease…” (H083) “… they don’t know as much about back pain and spondyloarthropathies, they wouldn’t necessarily know which test to ask for” (H082)
Sub-optimal practice in healthcare	Configuration of, and practice within, healthcare services slowing down journey to diagnosis	Time	“…clinicians don’t have enough time to spend with the patient, or consultants who make this diagnosis, or GPs who refer them or you know suspect the patient has AS, don’t have enough time to actually sort of look into those nuances to make a differential diagnosis” (H091) “GPs just need to be aware that they’re referring into the right service at the right time for the patient. And that’s a bit difficult if you’ve only got 5 or 10 min with somebody isn’t it…” (H071)
		Clinical guidance	“… it ends up that there’s quite a strong guidance to manage everything essentially as mechanical. … understandably it’s playing to the incidence and prevalence numbers and to this whole public health aspect that we don’t want to be overly irradiating people unnecessarily…”
		Referral issues	“… it probably took close on twelve months from first presentation to secondary care referral and then of course there’s some additional delay after referral to first consultant appointment” (H078) “Then you’ve got the delay in rheumatology seeing the patient. A lot of services are very stretched” (H072)
		“A bit of a revolving door”	“… they might have been seen by a service like ours before, been investigated, nothing maybe’s shown up at that point in time, so they’ve been discharged. And then it may be that they come back round or there’s been a bit of a revolving door until things are maybe a bit more clear with their symptoms” (H071) “… then we’re into potentially quite long primary care delay is going round that loop potentially a number of times…” (H078)
		Communication between HCPs	“… my direct line manager…. was traumatised when she found out I’d been emailing backwards and forwards with a consultant saying this is misdiagnosed. She said to me the protocol was that I had to go back to the GP, write to the GP for the GP to then question the diagnosis to my consultant, that was the standard operating procedure…” (H091)
Patient behaviour and characteristics	Patient behaviour and personality traits slowing down journey to axSpA diagnosis	Presenting to healthcare	“… even if you’re deliberately looking for it, and think, ‘okay, I’ll keep an eye on that patient’, and then they don’t reconsult within a couple of years, it’s kind of gone, and you might not see the same GP at that point anyway…” (H076) “… they just sort of say you know, just everyone gets back pain, it’s common, I didn’t go to my doctor I just sort of… it didn’t really stop me from doing anything…” (H083)
		Gender	“… there’s also gender bias I find, you know men tend to get diagnosed with AS quicker than females” (H091)
Patient/HCP interaction	Barriers caused by issues with the interaction between patients and the HCPs with whom they consult		“I find patients really struggle to answer stiffness questions and how you ask if something is stiff, it doesn’t really make sense to them, you might ask about night pain but actually not clarify well when is it in the night…” (H083) “…some health professionals I think stop hearing what they’re saying when they’re saying the same complaints over and over again” (H091)

#### AxSpA is difficult to diagnose

HCPs felt the difficulty of diagnosing axSpA was one of the main causes of delayed diagnosis. Two closely related sub-themes arose in this area of conversation:

1. AxSpA is difficult to define and differentiate, as many symptoms can overlap with other diseases and conditions, including fibromyalgia and mechanical musculoskeletal issues.
2. The outcomes of investigations into possible axSpA are not always clear, often requiring time and repetition before any degree of certainty can be attained.

#### Lack of awareness of axSpA

A lack of awareness of axSpA in patients and the general public was stated by many as a possible reason for delay in seeking consultation for their symptoms. In Addition, the majority of HCPs identified the lack of awareness of axSpA in healthcare as a cause of delay.

#### Sub-optimal practice in healthcare

It was frequently felt that the organisation of the healthcare system itself was a cause of diagnostic delay. The main problems identified were insufficient time for consultations, a lack of guidance and resources, and problems relating to referral and the ‘revolving door’ nature of healthcare, i.e. patients being bounced between departments to little productive end.

#### Patient behaviour and characteristics

HCPs observed that patients delayed presenting with their symptoms or absented themselves from healthcare for extended periods of time. This both intrinsically led directly to delay in diagnosis and also made continued investigation and observation far more difficult. It was also observed that where patients were ostensibly coping or able to attend work, they often did not consult regularly for their symptoms.

#### Patient/HCP interactions

HCPs described how patients found it difficult to articulate their symptoms in a way that created a clear picture. For example, there was a common conflation of describing pain and stiffness, where separating these two concepts may give a more accurate and useful understanding of a patient’s condition. HCPs also felt that there is an issue of dismissiveness among HCPs, partially caused by pain being such a common complaint and partially as some patients reconsult repeatedly with the same complaint leading to reduce receptivity from their doctor.

### The healthcare perspective of facilitators of diagnosis

Regarding facilitators of diagnosis of axSpA, four themes were identified from interviews with HCPs: promoting awareness of the disease in primary care and the general public, symptoms and characteristics that could raise suspicion of axSpA, improvements to practice in healthcare, and improved patient/HCP interactions (Table 5).

#### Promoting awareness of axSpA

HCPs identified the different ways that awareness of axSpA, could be promoted including via national charities, in hospital waiting rooms, in GP surgeries and through public campaigns.

In primary care there was recognition of the need to improve the education of GPs and AHPs and provide consultation tools to aid diagnosis.

#### Symptoms and characteristics that could raise suspicion of axSpA

All HCPs listed symptoms and characteristics of patients that would indicate a clinical suspicion of axSpA, tabulated below by order of frequency (Table 4). It was felt that if these were widely noticed in concert, suspicion could more readily be increased that a patient’s pain might be inflammatory in character, raising the possibility of axSpA.

**Table 4:** Suspicion-raising characteristics mentioned by number of HCPs

Symptom	HCP* mentions (%)
Lower back pain for 3 months or longer	13 (92.9)
Early morning stiffness	9 (63.3)
Symptom onset younger than 45 years	7 (50)
Back stiffness	6 (42.9)
Family history of SpA	6 (42.9)
History of inflammatory bowel disease	6 (42.9)
Psoriasis	6 (42.9)
Peripheral arthritis	6 (42.9)
Buttock pain	5 (35.7)
Family history of psoriasis	4 (28.6)
Symptoms respond to anti-inflammatories	4 (28.6)
Symptoms improving with exercise and movement	4 (28.6)

*Healthcare professional

#### Improving practice in healthcare

Clarifying the referral process for patients and improving the referral time were both seen as important ways of reducing diagnostic delay. Several HCPs felt that the availability of first contact physiotherapists at the beginning of clinical presentation might reduce diagnostic delay by allowing for a more detailed consultation. In Addition, HCPs using a focussed sets of exploratory questions could be employed when there is a clinical suspicion of inflammatory pain could improve the diagnosis of axSpA.

#### Improving patient/HCP interactions

Improving the effectiveness of interactions between patients and HCPs was seen to have scope to facilitate faster diagnosis of axSpA. Being open to patient input regarding their own symptoms and possible diagnosis was viewed as being important (Table 5).

**Table 5:** Healthcare perspective facilitators of diagnosis

Theme	Sub-themes	Sub-themes	Example quotes
Promoting awareness	Different means of raising awareness to speed up diagnosis times	Promoting awareness in the public	“… presenting three symptoms which in combination might raise concern and then suggesting talking to your doctor… so you see it on the back of buses… I suppose it would certainly trigger a patient’s concern… I’m sure you can place somebody in Coronation Street with it… that often for the short term triggers people to consult about their symptoms, if they’ve seen somebody else with them on telly or in the media…” (H089) “… we do have a patient group [in hospital] we are looking at getting televisions on the walls so we can start using promotional information on there around our service… in waiting areas when people are sitting there…” (H083)
		Promoting awareness in primary care	“… it’s got to fundamentally come down to GP awareness because that’s the patient’s first port of call… so it’s an educational role, I suppose to GPs and to primary care physiotherapists…” (H080) “… I think it’s important to educate the primary care practitioners and they know what questions to ask, if somebody came with back pain, you ask, this question, if that then refer… we want to make their life easier don’t we?” (H091)
		Education delivery	“… it is that training need and not necessarily just a one off training need, but that refresher maybe every couple of years just to keep it fresh in the minds of those in primary care so they don’t forget some of the things that they maybe learned earlier in their career.” (H071) “I think there could be initiatives linking in with primary care clinicians, education and workshops to discuss more and improve knowledge and competence around diagnosing axial SpA…” (H072)
Symptoms and characteristics that could raise suspicion of axSpA	Symptoms and characteristics that could raise clinical suspicion of axSpA		“I would look at lower back pain, inflammatory in nature so we’re talking about waking in the night usually the second part of the night, pain that’s worse in static conditions.” (H071) “Their age and symptoms starting from an early age and with non specific mechanisms of onset of symptoms, so no injury, no trauma. Alternating buttock pain is another one that would make me think…” (H082)
Improving practice in healthcare		The referral process	“… optimising the referral pathways for patients so that we have a streamlined process where patients with axial SpA are seen in a timely fashion, they get scans and diagnostic testing in a timely fashion.” (H072) “… it would be helpful… having fast track pathways where there’s a dedicated clinic for the patients who are not coming to a general pool. And if we can segregate that pool of patients who are waiting to be diagnosed, whether they’ve got axial spondyloarthritis, then direct them into a particular clinic, we can segregate the patients into different categories, for ones who have definitely not got axSpA can be discharged, category B has definitely got the disease…” (H074) “Referral to a specialist centre earlier on to break this revolving door really of GP to physio to GP to physio…” (H083)
		Improvements to first contact	“… first-contact physiotherapists or other, working as an integral part of a primary care team in the same way that midwives and district nurses do, so that referrals from the GP to the physio could happen with reasonable rapidity…” (H078)
		Enhancing consultations	“… you ask a patient, you know under 45, under 35, if they’re waking in the middle of the night feeling painful, stiff symptoms in their back, if they get on and off back pain, if it improves in movement, there’s a few other symptoms you can look for… so in a normal GP consultation for example, I think it could be ascertained quite quickly…” (H076) “… you could, within the search that causes that pop up to trigger, put in things like age and consultation patterns and high NSAID use or associated stiffness, have you considered this as a possibility and consider a referral to a rheumatologist…” (H089)
Patient/HCP interactions	How communication and interaction between HCPs and patients could be improved to speed diagnosis		“I would tend to ask what you were like growing up, were you sporty and they go oh yeah I didn’t have any problems then, what about college, uni, work, that sort of thing… I kind of ask through their phases of life… I don’t think people actually tend to give that kind of information freely…” (H083) “If there really is an antipathy then there’s a communication failure. Occasionally clinicians are unreasonable, occasionally patients are unreasonable but in general if there’s a sense of antipathy, it’s a sign the consultation needs to begin again … I think there would be some patients and clinicians who would be willing to almost literally do that, to start the consultation again. Sometimes it means the patient will go off and see another clinician…” (H078)

## Discussion

This study explored barriers and facilitators to the diagnosis of axSpA from the perspectives of both patients with axSpA and HCPs. Regarding barriers to diagnosis, there was a notable concordance between patients with axSpA and HCPs regarding; ineffective communication, difficulties diagnosing axSpA, sub-optimal practice in healthcare, lack of awareness and comprehension of the disease and patient behaviours. There was some agreement regarding facilitators of diagnosis, such as education and awareness campaigns, positive communication, and patient advocacy, improving referral pathways and identifying means of raising suspicion of inflammatory arthritis earlier (Box 1). One divergence between the two groups regarding facilitators is that some patients regarded “serendipity” as having facilitated their diagnosis, reflecting the position that the healthcare system may not be identifying patients systematically in its current arrangement.

**Box 1:** Top level themes

	Patients	HCPs
Barriers to Diagnosis	Patient/HCP interaction AxSpA is difficult to diagnose Patient behaviour Lack of awareness of axSpA Sub-optimal practice in healthcare	AxSpA is difficult to diagnose Lack of awareness of axSpA Sub-optimal practice in healthcare Patient behaviour and characteristics Patient/HCP interaction
Facilitators of Diagnosis	Patient behaviour and advocacy Patient characteristics Good practice in healthcare Education and awareness Serendipity	Promoting awareness Symptoms and characteristics that could raise suspicion of axSpA Improving practice in healthcare

All participants were aware of the difficulty of differentiating between axSpA and other conditions which can cause similar symptoms, including osteoarthritis and fibromyalgia. Lack of awareness in HCPs was perceived to cause greater delay compared to low awareness in the general public. Low awareness of axSpA in Dutch GPs, contributed to missing many possible cases [12]. It may also be the case that low awareness and understanding of the disease feeds into many of the issues of communication; if a GP is unable to link the associations within a collection of interacting symptoms, they may focus on the most obvious, such as pain. This understandable approach reflects the high frequency of pain encountered in community healthcare, with only a comparatively low proportion being caused by axSpA [27]. In this study the lack of further exploration/investigation can be perceived by the patient as dismissal, and GPs can perceive the patient’s persistence as an inconvenience rather than a suggestion of recurrent symptoms; these findings are not unique and have previously been described in research exploring GP consultations [28].

Education and awareness of axSpA was seen by many patients and HCPs to be an important means of reducing diagnostic delay. Since 2013, the National Rheumatoid Arthritis Society (NRAS) [29] have coordinated the Rheumatoid Arthritis Awareness Week, with the aim of increasing awareness of rheumatoid arthritis [30]. The National Axial Spondyloarthritis Society’s (NASS) ‘Act on axial SpA’ campaign is currently focussed on increasing awareness of axSpA with the aim of reducing time to diagnosis; it utilises the acronym “SPINE” as a means of quickly conveying core concepts of axSpA: Symptoms starting slowly, Pain in the lower back, Improves with movement, Night time waking, Early onset [31]. However, this program was only launched recently, so quantifiable benefits have yet to be shown.

Specific education of HCPs would need to focus on three key areas: First, to be cognisant of communication style, such as outlined by NHS England in 2021 [32]; Secondly, not to ignore musculoskeletal pain in younger people, as it might be significant of something more complex. Thirdly, a family history of SpA is more common among people with axSpA [33], as is uveitis [34], psoriasis [35] and inflammatory bowel disease [36]. As primary healthcare is characterised by extreme time and financial pressures [37], any education would need to compliment and not increase workload demands.

Several HCPs felt that a simple set of questions based on the presence of factors more specific to axSpA, such as stiffness in the morning, waking in the second half of the night with pain, and buttock pain would be a useful diagnostic tool. Baraliakos et al. [38] in 2020 described using a short series of questions/investigations to identify individuals with potential axSpA requiring specialist review, as a refinement to Braun et al. [39], which comprise the current axSpA screening in NICE guidelines [20]. However, the Baraliakos referral strategy requires validation in an independent cohort and has not been tested in a UK population. In Addition, due to limitations in the sample size, there are residual questions regarding sensitivity and specificity of the described factors’ predictive capabilities.

Advocacy for patients with axSpA was an important factor contributing towards instigating the process of diagnosis; by taking friends and family to consultations to provide emotional support and assisting with communication with HCPs. Self-advocacy was strongly espoused by some patient participants, by being assertive in communications with clinicians. Many patients with axSpA found the most helpful HCPs were often the ones willing to acknowledge when they had reached the bounds of their knowledge and would seek further opinions or refer the patient. HCPs who didn’t underestimate the patients’ symptoms or those who displayed a pro-active attitude to problem solving were described in positive terms by patients. Patient advocacy has been described elsewhere as an essential facilitator of diagnosis, not just as an aid to communication, but also to ensure the patient felt they were consulting with an HCP who met their information and care needs [11]. One possible practical aid to patients’ self-advocacy and communication with their HCP would be easily accessible and understandable written material available in different formats (online/offline) which could give patients a guide on how to maximise the effectiveness of their consultation.

The fact that many patients with axSpA credited the success of their diagnosis to serendipity may be related to several possible factors. The first is that as this study recruited most of its patient participants through social media and respondents to NASS newsletter advertisements, there may be a selection bias towards individuals with more dramatic experiences. Another possibility is that serendipity, as presented by the participants of this study, is an artefact of post-hoc narrative creation, in which it played less of a genuine part in diagnosis than was represented. A final possibility is that patients who experience longer delays are comparatively more likely to experience chance encounters/circumstances which lead to diagnosis. Serendipity or serendipity as facilitators are not commonly referenced in scientific literature, possibly due to their relative unquantifiability, but are not entirely absent. One study into pathways of care and diagnosis for children with vascular malformations presents serendipity as a facilitator of diagnosis, specifically linking it to knowing or encountering specific individuals [40]. In the present study, this definition holds; patients who felt serendipity facilitated their diagnosis often described chance encounters with people who were able to identify their symptoms.

The findings of this study have some notable implications for clinical practice. Awareness and understanding of axSpA needs to improve within the healthcare system and among the general public, as does communication with patients. In Addition, the time from first primary care consultation and referral to secondary care needs to reduce, and the process of referral clarified. Not only would this streamline diagnosis, but it may also keep patients more motivated to actively seek diagnosis of their own volition. Martindale et al. [10] found, as with the present study, that patients described poor interactions with healthcare as a factor that demotivated them in their search for answers about their symptoms.

Many participants stated they felt the time available for GP consultations was insufficient to communicate their symptoms effectively. Unfortunately, while increasing the time of GP consultations is desirable, it may not be realistic due to pressures on the UK healthcare system. However, as back pain is increasingly being managed by primary care-based physiotherapists this service could be leveraged to improve early consultations [41]. Where implementation of first contact physiotherapists (FCPs) has been undertaken, results have been encouraging, with patients showing high confidence in FCPs [42]. In Addition, the majority of patients who see an FCP do not then need a repeat consultation with their GP, demonstrably reducing the burden on GPs [42]. Underpinning all these points is the need for greater education for HCPs regarding axSpA.

The main strength and novelty of this study is that it is the first qualitative study in the UK to collect data in parallel, from both patients with axSpA and HCPs, regarding barriers and facilitators in diagnosing axSpA. The results show a consensus between patients and HCPs on the need to improve prompt diagnosis. A limitation of this work relates to the composition of the sample, which included more women than men. Though this may influence aspects of our results, this imbalance is perhaps not surprising when women have traditionally experienced more delay due to misunderstanding of their disease [43]. Future research will benefit from including a better balance of male and female patients as it has been noted that the disease does manifest in and affect sexes differently [43]. Disease presentation and experiences throughout the patient sample were highly varied, meaning that while a wide range of experiences were represented, the potential for external validation is limited. Socioeconomic status and ethnicity were not recorded in this study, and both could influence the experience of the disease. Furthermore, the relatively small size and heterogeneity of the HCP sample, while representing a cross-section of healthcare, may also limit the extent to which the findings of this study can be externally validated. Future research in this area with purposive sampling for greater diversity would reflect the views of a wider section of the populace.

## Conclusion

This study has identified the need to improve patient and HCP education and raise the awareness of axSpA. In Addition, improving the quality of interaction at initial contact with healthcare through patient advocacy, effective communication (including active listening and admitting when a solution is elusive and needs further input) and utilisation of primary care physiotherapists may help alleviate avoidable delay. Finally, clearer identified referral pathways that are quick and effective to use are necessary to reduce the “bouncing about” between services experienced by patients, therefore reducing diagnostic delay for axSpA.

### Supplementary Information

Below is the link to the electronic supplementary material.

Supplementary file1 (PDF 481 KB)

Supplementary file2 (DOCX 27 KB)

Supplementary file3 (DOCX 16 KB)

Supplementary file4 (DOCX 15 KB)

Supplementary file5 (DOCX 15 KB)

## Data Availability

Illustrative data for this study have been provided in the main manuscript and in addition to being the best representation of the themes which arose from the study analysis, they were selected and necessarily submitted to redaction to protect the identities of involved participants. Due to the small sample-size of this study, to protect against identifiability the original datasets, i.e. the full interview transcripts, will not be made publicly available. In addition to the concern about identifiability, conversations which took place during these semi-structured interviews occasionally addressed concerns relevant to the narrative of a participant's diagnostic journey, but are not appropriate to address here as they do not contribute directly to answering the identified research question within the research remit for which participants consented for their data to be used. For this further reason, access to the original transcripts is not appropriate.
