# Supplementary material for: Barriers and facilitators in diagnosing axial spondyloarthritis: a qualitative study
Source: Rheumatol Int. 2024 Mar 12;44(5):863–84. doi: 10.1007/s00296-024-05554-z (PMC10980652; doi:10.1007/s00296-024-05554-z)
Supplement: Supplementary file 2 — Supplementary file2 (DOCX 27 KB) [file 296_2024_5554_MOESM2_ESM.docx]

**Topic Guide Interview, 30-60 minutes**

***Interview Objectives***

**Primary Aim:** To gain understanding of the barriers to and facilitators of diagnosis of axial spondyloarthritis (axSpA).

OR

Understanding reasons for diagnostic delay in axSpA

**Sample**

Between 15 and 20 patients will be interviewed over the telephone for this study. The patients will be recruited from 1) the membership of the National Ankylosing Spondylitis Society or 3) axSpA patients answering an announcement made on social media.

**Selection criteria**

- English speaking
- Cognitively capable of participating in and emotionally coping with the interview
- Diagnosed with axial spondyloarthritis
- Subject to diagnostic delay of greater than 12 months

**Introductions, consent**

- Introductions. I am a PhD student and don’t have a clinical background.
- Inform patient that the session is to be recorded and remind them that their testimonial will be anonymised during the transcription process.
- Confirmation that the patient has read and become acquainted with the patient information sheet (PIS) and consents to take part in the study.
- Inform patient that the interview will take up to an hour and what the structure of the interview will be.
  - Background information
  - Exploration of patient’s understanding of axSpA and diagnostic delay
  - Patient’s experience of axSpA and diagnostic delay
  - Exploration of patient’s opinions regarding axSpA and diagnostic delay
- Reiteration of the content of the PIS to ensure understanding.

**Background Information, introducing research**

- Description of axSpA: Axial spondyloarthritis, previously and also known as ankylosing spondylitis
  - - For the most part I’ll call it axial spondyloarthitis and axSpA
  - an inflammatory arthritis found in around 1 in 200 people.
  - Primarily affects the lower back, causing chronic back pain and changes in the spine which can lead to reduced range of ability.
  - Patients still suffer between two and eight years diagnostic delay, sometimes far more.
- **This research is part of a three part project looking to assist in the reduction of diagnostic delay**
  - The first part is a review of all research into diagnostic delay to find out global levels of delay
  - After this current study, another will be run looking at the relevant medical histories of patients leading up to their diagnosis of axSpA
- This interview based study aims to find out about patient and HCP experience of the axSpA journey to diagnosis
- We want to know more about what holds up diagnosis and where possible, what speeds it up.
- Do you have some questions at this stage?

**Understanding of axSpA and diagnostic delay**

- Knowledge of axSpA, its delay, the outcomes of its delay
  - Before what I just told you, what would you have thought the amount of delay was for the disease?
  - Are you aware of the outcomes of delayed diagnosis?

**Experience**

- How long did it take to reach your diagnosis of axSpA?
  - Are you aware of what was identified as your “initial symptom of onset”?
  - Do you agree with that assessment?
- What symptoms did you first present with and to whom?
- What did you think was causing these symptoms?

*Motivators for and Experience of First Consultation*

- What motivated you to go and see your GP?
  - How long had you had your symptoms before you went to see your GP?
  - Were you motivated by the experiences or opinions of other patients with axSpA?
- What were you hoping the GP would do (expectations of the consultation)?
- Can you recall what happened when you went to see your GP?
- Did your GP suggest to you what might have caused your symptoms?
- What did your GP advocate trying to help with your symptoms?

*Referral and Process of Diagnosis*

- Did you have to go for any investigation?
- How long had you had your symptoms before you were given a diagnosis?
- How did it feel when diagnosis was reached?

*Barriers/Facilitators*

- Did you encounter any setbacks to diagnosis?
  - Uncertainty regarding cause of symptoms
  - Mis-diagnosis
  - Personal circumstances
  - Referral delays
- Did you encounter anything which notably sped up your diagnosis?
  - Suspicion based on experience
  - Obvious set of circumstances Immediate or fast referral
  - Which stages of medical care did you feel were the quickest and most efficient?
- Any knowledge of the experiences of other axSpA patients?

**Opinions**

- On the road to receiving your diagnosis, what processes could have been improved?
  - This includes your own experience prior to consultation with any healthcare professional regarding your arthritis
  - Process of referral
  - Certainty of diagnosis
- What ideas do you have for how diagnostic delay could be reduced for axial spondyloarthritis?
  - What elements of the diagnostic process do you find the most important?
  - What would you respond to most readily (advertising, press, warnings in GPs surgery etc)?
  - What could be altered on the HCP side to better facilitate timely diagnosis?
  - How would this apply to yourself?
  - How would this apply to the wider population?

That brings us to the end of the interview! Thank you so much for your time, it’s very much appreciated. Your input and insights have been very valuable and will make up part of a study which should hopefully prove helpful to lots of people either diagnosed with or managing axSpA in the future.

If you want, I’ll keep you appraised of the results of this study and the ways in which these results will be used in the future.

Do you have some final questions?

**Topic Guide Healthcare Professionals Interview, 30 minutes**

***Interview Objectives***

**Primary Aim:** To gain understanding of the barriers and facilitators of diagnosis of axial spondyloarthritis (axSpA).

**Sample**

Healthcare professionals (HCPs) of different specialities will be recruited for interview (rheumatologists, GPs, general practice nurses, physiotherapists, other allied health professionals). Recruitment of HCPs is purposive, with HCPs managing axSpA being targeted for inclusion.

**Selection Criteria**

- English speaking
- Currently or previously Managing axSpA
- Operating within the West Midlands area

# Introductions, consent

- Introductions. I am a PhD student and don’t have a clinical background.
- Inform interviewee that the interview is being recorded and that they will not be identifiable after transcription.
- Confirmation that the participant has read and become acquainted with the participant information sheet (PIS) and consents to take part in the study.
- Inform interviewee that the interview will take between 30 minutes and an hour, confirm that this time is currently available.
- **This study makes up part of my PhD, which also involves a systematic review and a case-control study**
  - This interview based study aims to find out about patient and HCP experience of the axSpA journey to diagnosis
  - We want to know more about what holds up diagnosis and where possible, what speeds it up.
- Outline structure and timing of interview
  - The interview is designed to last between half an hour to an hour
  - Structure
    - Background information
    - Information about the interviewee and their knowledge of axSpA
    - Experience of managing axSpA
    - Experience and perception of axSpA patient experience
    - Conversation on how to help reduce delay

# Background Information, introducing research (time – no more than a minute)

I have a brief section to catch anyone who needs it up on axSpA – do you need a run through on prevalence, characteristic and diagnostic delay, or shall we skip straight to the interview?

- ***Apologies to those who already know this, perhaps cut altogether if they already have good grounding***:
  - Axial spondyloarthritis, previously and also known as ankylosing spondylitis
    - For the most part I’ll call it axial spondyloarthitis and axSpA
  - an inflammatory arthritis found in around 1 in 200 people.
  - Primarily affects the lower back, causing chronic back pain and changes in the spine which can lead to reduced range of ability.
  - Patients still suffer between two and eight years diagnostic delay, sometimes far more.
- Do you have some questions?

# Participant Introduction

- Now I’ve been introduced, along with the study, I’d like to hear a bit about yourself.
- We’ll talk at greater length about your experiences with axSpA, but briefly could you outline your specialty and work within it, and how this brings you into contact with axSpA?

# Experience of managing axSpA

- What symptoms do you associate with axSpA?
  - Can you imagine what you’d see in a patient to make you suspect axSpA?
- How frequently do you encounter patients with symptoms suggestive of axSpA?
  - When did you last see a patient you thought might have axSpA?
    - What were they in for?
  - Characteristics of patients
  - Where referred from?
  - Where referred to?
- Can you think of a “typical” experience of the diagnostic process from HCP perspective?
  - What can you say about a typical referral process?
  - How long has often passed between a patient getting symptoms and going to their doctor?
  - How long do referrals take?
    - How are these prioritised?
- If you manage or treat axSpA, how would you treat/manage a patient who you suspected of having axSpA?
- What is your awareness of other HCP and AHP experiences of the axSpA management?
- Are there any resources or experts you call upon when assessing a patient with axSpA?
  - Guidelines?
  - Colleagues?
  - Experts?
  - Personal experience?
  - Social media?
- If you do use guidelines, how useful do you find these in real-world axSpA cases?
- **What we know so far is that this condition generally takes 8 years to be diagnosed**
- Why do you think that might be?
  - Patient behaviours
  - Unusual presentation
  - Complex morbidity
  - HCP related issues
  - Imaging
- Can you think of anything that would be in your power to assist in earlier diagnosis?

# Experience and Perception of patient experience of axSpA

- Generally, how do you think patients experience the diagnostic journey for axSpA?
  - Have you seen patient experiences differing based on their referral journey?
  - Have you seen patient experiences differing based on their reasons for first seeking help?
- Up until the point of diagnosis, what do patients on the diagnostic journey want to talk about the most?
  - Do patients get enough time to discuss their symptoms in enough depth to suggest a less obvious diagnosis such as axSpA?
  - Process things?
  - Symptom things?
  - Access?

**IF NOT COVERED EARLIER:
Experience of personal and observed levels of guideline implementation**

- Awareness of NICE guidelines?
- To what extent do you feel NICE guidelines are adhered to?
  - Is adherence to NICE guidelines all the time realistic?
  - Are alternative routes sometimes necessary?
- If aware of ASAS assessment criteria: have things improved since their introduction?
- If unaware of ASAS assessment criteria: do you perceive the process of diagnosis to have improved in recent years?

# Opinions regarding what could help with diagnostic delay

- Generally, how could things be improved in the diagnostic process?
  - What has been enacted in other areas which has improved diagnosis times?
  - What do you think regarding the feasibility of a campaign similar to that of stroke and heart attacks which gave people a more standardised vocabulary with which to communicate symptoms with their HCP?

# Interview End

Thank you so much for your involvement today, your input and insight has been extremely helpful and enlightening.

I understand your time is very precious and I’m very grateful for you being able to fit this in. I’ll be in touch if you want to know the outcomes of the study.
