# Supplementary material for: Barriers and facilitators in diagnosing axial spondyloarthritis: a qualitative study
Source: Rheumatol Int. 2024 Mar 12;44(5):863–84. doi: 10.1007/s00296-024-05554-z (PMC10980652; doi:10.1007/s00296-024-05554-z)
Supplement: Supplementary file 3 — Supplementary file3 (DOCX 16 KB) [file 296_2024_5554_MOESM3_ESM.docx]

Thematic analysis was employed as the main method of analysing the study data, based upon a phenomenological framework. The process of thematic analysis used in this study, was based on the steps described by Braun and Clarke (2006) as follows:

1. **Familiarisation with data: transcription of interviews, repeated reading and noting of initial codes**
   Familiarisation began prior to the transcription process, during the interviews. This did not extend to formal coding but involved exploratory thoughts and hypotheses regarding the content of the interview. Turns of phrase were noted. Further salient details were also noted for context.
   Six interviews (3 patients, 3 HCPs) were transcribed by myself, which provided another layer of familiarisation. Interviews were read over several times before what could be considered early, non-formalised coding was undertaken using highlighter and annotations on paper printouts and pdfs interacted with on an iPad.
2. **Generation of initial codes: coding interesting and relevant features from the interviews**
   The transcripts were imported into NVivo 12 and coding began using this software. Coding at this stage was exhaustive and not only limited to direct discussion of barriers to and facilitators of axSpA diagnosis. All information regarded as salient was coded, along with more ancillary information regarding lived experiences; this was due to the suspicion that on further exploration, the more outlying information would at the very least provide further context for the “main” narrative, i.e., that regarding barriers and facilitators in diagnosis.
3. **Identifying themes: examining the coded data to identify specific patterns of meaning**
   At this stage, within NVivo, the codes identified in the previous stage were collated into hierarchies, based on how closely related they were.
4. **Review of themes: checking that the themes represent the data and address the research question, along with the generation of a thematic map**
   The identified themes were then reviewed in two phases. The first phase involved reviewing the codes collated into themes to see whether the codes contained within themes belonged there and reached a level of coherence with each other sufficient to be considered genuinely relevant to each other. If this was not the case, codes were removed from the theme and placed elsewhere.
   In the second phase themes were reviewed to ensure they contributed to answering the research question and whether they could justify their existence as discrete themes. Some themes were amalgamated into each other, as they overlapped to such an extent that their boundaries were arbitrary. Some themes were split into two, and some were renamed to better represent their constituent codes and to relate more closely to the research questions.
5. **Defining and naming themes: refining the specifics of each theme. Clear names for themes were derived**
   This stage involved formalising the themes, ensuring they contributed to answering the research question and the names of themes were self-explanatory and descriptive of constituent codes.
6. **Writing up: final analysis of data, discussion of analysis and full writing up of study**
