# Supplementary material for: Barriers and facilitators in diagnosing axial spondyloarthritis: a qualitative study
Source: Rheumatol Int. 2024 Mar 12;44(5):863–84. doi: 10.1007/s00296-024-05554-z (PMC10980652; doi:10.1007/s00296-024-05554-z)
Supplement: Supplementary file 4 — Supplementary file4 (DOCX 15 KB) [file 296_2024_5554_MOESM4_ESM.docx]

Supplementary Table 4: Characteristics of patient sample

| ID | Gender | Year of Diagnosis | Age at Interview | Age at Symptom onset | Age at Diagnosis | Diagnostic Delay |
| --- | --- | --- | --- | --- | --- | --- |
| P002 | F | 2016 | 54 | 25 | 50 | 25 |
| P003 | M | 2017 | 42 | 27 | 39 | 12 |
| P004 | M | 2011 | 43 | 20 | 34 | 14 |
| P010 | F | 2011 | 40 | 15 | 32 | 17 |
| P015 | F | 2017 | 43 | 20 | 40 | 20 |
| P018 | F | 2019 | 51 | 30 | 50 | 10 |
| P021 | F | 2018 | 29 | 20 | 27 | 7 |
| P024 | F | 2020 | - | - | - | 13 |
| P025 | F | 2013 | 47 | 12 | 40 | 20 |
| P030 | F | 2019 | 54 | 30 | 53 | 21 |
| P032 | F | 2010 | 30 | 11 | 20 | 9 |
| P033 | F | - | - | - | - | 20 |
| P035 | M | 2016 | 24 | 19 | 20 | 1 |
| P035 | M | 2006 | 59 | 25 | 45 | 20 |
