# Supplementary material for: Barriers and facilitators in diagnosing axial spondyloarthritis: a qualitative study
Source: Rheumatol Int. 2024 Mar 12;44(5):863–84. doi: 10.1007/s00296-024-05554-z (PMC10980652; doi:10.1007/s00296-024-05554-z)
Supplement: Supplementary file 5 — Supplementary file5 (DOCX 15 KB) [file 296_2024_5554_MOESM5_ESM.docx]

Supplementary Table 5: Characteristics of healthcare professional sample

| ID | Gender | Specialism | Frequency of seeing patient with suspected axSpA |
| --- | --- | --- | --- |
| H071 | F | Musculoskeletal extended scope physiotherapist | “… you’re probably looking at one every couple of months.  So a handful a year…” |
| H072 | M | Rheumatologist | Wasn’t able to specify |
| H074 | M | Rheumatologist | Wasn’t able to specify |
| H075 | M | Rheumatologist | “I would probably say six or seven [per week]… incidence  is a bit less… ten [new patients] in six months…” |
| H076 | M | Physiotherapist | “… I definitely say monthly… I would usually have some AS patients on my caseload…” |
| H077 | M | GP | “… in the past 12 months… maybe 2 or 3 who’ve been  formally diagnosed with axSpA…” |
| H078 | M | GP | “Very infrequently… I would suspect it no more than  a couple of times a year…” |
| H079 | M | GP | “I’m sure there should be somebody at least once a month  or a couple of times a month…” |
| H080 | F | Physiotherapist | “… probably there’s going to be about 2 or 3 a month…” |
| H082 | F | Spinal service physiotherapist | “… about 5% [of patients presenting with back pain].” |
| H083 | F | Spinal service physiotherapist | “… up to a dozen a year…” |
| H085 | F | Specialist nurse | “… it’s probably about 40% of our biologic patients…” |
| H089 | F | GP | “… I could probably think of three times in the past couple of years or so…” |
| H091 | F | Occupational therapist | “… it’s hard to come up with a sensible number really.” |
